# Under-approximating loops in C programs for fast counterexample detection

**DOI:** 10.1007/s10703-015-0228-1

**Published:** 2015-04-17

**Authors:** Daniel Kroening, Matt Lewis, Georg Weissenbacher

**Affiliations:** University of Oxford, Oxford, UK; Vienna University of Technology, Vienna, Austria

**Keywords:** Model checking, Loop acceleration, Underapproximation, Counterexamples

## Abstract

Many software model checkers only detect counterexamples with deep loops after exploring numerous spurious and increasingly longer counterexamples. We propose a technique that aims at eliminating this weakness by constructing auxiliary paths that represent the effect of a range of loop iterations. Unlike acceleration, which captures the exact effect of arbitrarily many loop iterations, these auxiliary paths may under-approximate the behaviour of the loops. In return, the approximation is sound with respect to the bit-vector semantics of programs. Our approach supports arbitrary conditions and assignments to arrays in the loop body, but may as a result introduce quantified conditionals. To reduce the resulting performance penalty, we present two quantifier elimination techniques specially geared towards our application. Loop under-approximation can be combined with a broad range of verification techniques. We paired our techniques with lazy abstraction and bounded model checking, and evaluated the resulting tool on a number of buffer overflow benchmarks, demonstrating its ability to efficiently detect deep counterexamples in C programs that manipulate arrays.

## Introduction

The generation of *diagnostic counterexamples* is a key feature of model checking. Counterexamples serve as witness for the refutation of a property, and are an invaluable aid to the engineer for understanding and repairing the fault.

Counterexamples are particularly important in software model checking, as bugs in software frequently require thousands of transitions to be executed, and are thus difficult to reproduce without the help of an explicit error trace. Existing software model checkers, however, fail to scale when analysing programs with bugs that involve many iterations of a loop. The primary reason for the inability of many existing tools to discover such “deep” bugs is that exploration is performed in a breadth-first fashion: the detection of an unsafe execution traversing a loop involves the repeated refutation of increasingly longer spurious counterexamples. The analyser first considers a potential error trace with one loop iteration, only to discover that this trace is infeasible. As consequence, the analyser will increase the search depth, usually by considering one further loop iteration. In practice, the computational effort required to discover an assertion violation thus grows exponentially with the depth of the bug.

Notably, the problem is not limited to procedures based on abstraction, such as predicate abstraction or abstraction with interpolants. Bounded model checking (BMC) is optimised for discovering bugs up to a given depth $k$, but the computational cost grows exponentially in $k$.

The contribution of this paper is a new technique that enables scalable detection of deep bugs. We transform the program by adding a new, auxiliary path to loops that summarises the effect of a parametric number of iterations of the loop. Similar to acceleration, which captures the exact effect of arbitrarily many iterations of an integer relation by computing its reflexive transitive closure in one step [4, 8, 11], we construct a summary of the behaviour of the loop. By symbolically bounding the number of iterations, we obtain an *under-approximation* which is sound with respect to the bit-vector semantics of programs. Thus, we avoid false alarms that might be triggered by modeling variables as integers.

In contrast to related work, our technique supports assignments to arrays and arbitrary conditional branching by computing quantified conditionals. As the computational cost of analysing programs with quantifiers is high, we introduce two novel techniques for summarising certain conditionals without quantifiers. The key insight is that many conditionals in programs (e.g., loop exit conditions such as $$\mathtt{i}\le
								100$$ or even $$\mathtt{i}\ne
								100$$) exhibit a certain monotonicity property that allows us to drop quantifiers.

Our approximation can be combined soundly with a broad range of verification engines, including predicate abstraction, lazy abstraction with interpolation [19], and bounded software model checking [5]. To demonstrate this versatility, we combined our technique with lazy abstraction and the Cbmc  [5] model checker. We evaluated the resulting tool on a large suite of benchmarks known to contain deep paths, demonstrating our ability to efficiently detect deep counterexamples in C programs that manipulate arrays.

## Outline

### Notation and preliminaries

We restrict our presentation to a simple imperative language comprising assignments, assumptions, and assertions. A program is a control flow graph $$\langle
									V,E,\lambda \rangle $$, where $$V$$ and $$E$$ are sets of vertices and edges, respectively, and $$\lambda $$ is a labelling function mapping vertices to statements. Procedure calls are in-lined and omitted in our presentation. The behaviour of a program is defined by the paths in the control flow graph (CFG). A path $$\pi $$ of length $$m$$ is a sequence of contiguous edges $$e_1\,e_2 \ldots e_m$$ ($$e_i\in E$$, $$1\le i\le m$$). Abusing our notation, we use the corresponding sequence of statements $$\lambda (e_1)\mathtt{;}\,\lambda
									(e_1)\mathtt{;}\ldots \lambda (e_m)$$ to represent paths (where ; denotes the non-commutative path concatenation operator). We use $$\varepsilon $$ to denote the path of length $$0$$ and inductively define $$\pi ^n$$ as $$\pi ^0=\varepsilon $$ and $$\pi ^{n+1}=\pi ^n\mathtt{;}\,\pi
									$$ (for $$n\ge 0$$). In accordance with [20], 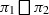 represents the non-deterministic choice between two paths, i.e., 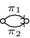. The commutative operator 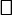 is extended to sets of paths in the usual manner. 

We use first-order logic (defined as usual) with background theories commonly used in software verification (such as arithmetic, bit-vectors, arrays and uninterpreted functions) to represent program expressions and predicates. $${\mathsf {T}}$$ ($$\mathsf {F}$$) represents the predicate that is always true (false). We use * to indicate non-deterministic values. The semantics for statements and paths is determined by the predicate transformers in Table 1 (see [20]). A Hoare triple $$ \left\{ {P}\right\} \;{\pi }\;\left\{
									{Q}\right\} $$ comprises a pre-condition $$P$$, a path $$\pi $$, and a post-condition $$Q$$ such that $$ sp({\pi },{P}) $$ implies $$Q$$. Given a set $$\mathtt{X}=\{\mathtt{x}_1,\ldots
									,\mathtt{x}_k\}$$ of $$k$$ variables, we introduce corresponding sets $${}^{\backprime }\mathtt{X}=\{{}^{\backprime
									}\mathtt{x}_1,\ldots ,{}^{\backprime
									}\mathtt{x}_k\}$$ and $$\mathtt{X}^{\prime }=\{\mathtt{x}^{\prime
									}_1,\ldots ,\mathtt{x}^{\prime }_k\}$$ of *primed* variables to refer to variables in prior and subsequent time-frames, respectively (where the term *time-frame* refers to an instance of $$\pi $$ in $$\pi ^n$$). We use $$\mathtt{X}^{\langle {i}\rangle
									}=\{{\mathtt{x}}^{\langle {i}\rangle }_1,\ldots
									,\mathtt{x}^{\langle {i}\rangle }_k\}$$ to refer to the variables in a specific time-frame $$i$$. The transition relation of $$\pi $$ is the predicate $$\lnot wlp\left( {\pi },{\bigvee _{i=1}^k
									\mathtt{x}_i\ne \mathtt{x}'_i }\right)
									$$ [20] and relates variables of two time frames (for example, for $$k=2$$ and the path $$\pi
									=[\mathtt{x_1}<0];\,\mathtt{x}_1=\mathtt{x}_2+1$$ we obtain $$(\mathtt{x}_1<0)\wedge
									(\mathtt{x}^{\prime }_1=\mathtt{x}_2+1)\wedge ({x}^{\prime
									}_2=\mathtt{x}_2)$$).

**Table 1:** Predicate transformers for simple program statements and paths

Path	Strongest postcondition	Weakest liberal precondition
$\pi$	$sp({\pi },{P})$	$wlp\left( {\pi },{Q}\right)$
$\varepsilon$ / skip	$P$	$Q$
x:= $e$	$\exists {}^{\backprime }\mathtt{x}\,.\,(\mathtt{x}=e[\mathtt{x}/{}^{\backprime }\mathtt{x}])\wedge P[\mathtt{x}/{}^{\backprime }\mathtt{x}]$	$Q[\mathtt{x}/e]$
x:=*	$\exists {}^{\backprime }\mathtt{x},v\,.\,(\mathtt{x}=v)\wedge P[\mathtt{x}/{}^{\backprime }\mathtt{x}]$	$\forall v\,.\,Q[\mathtt{x}/v]$
[$R$]	$P\wedge R$	$R\Rightarrow Q$
assert( $R$ )	$P\wedge R$	$R\Rightarrow Q$
$\pi _1$ ; $\pi _2$	$sp({\pi _2},{ sp({\pi _1},{P})})$	$wlp\left( {\pi _1},{ wlp\left( {\pi _2},{Q}\right) }\right)$
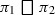	$sp({\pi _1},{P}) \vee sp({\pi _2},{P})$	$wlp\left( {\pi _1},{Q}\right) \wedge wlp\left( {\pi _2},{Q}\right)$

$Q[\mathtt{x}/e]$ denotes that all free occurrences of x in $Q$ are replaced with the expression $e$

### A motivating example

A common characteristic of many contemporary symbolic software model checking techniques (such as counterexample-guided abstraction refinement with predicate abstraction [1, 10], lazy abstraction with interpolants [19], and bounded model checking [5]) is that the computational effort required to discover an assertion violation may increase exponentially with the length of the corresponding counterexample path (c.f. [16]). In particular, the detection of assertion violations that require a large number of loop iterations results in the enumeration of increasingly longer *spurious* counterexamples traversing that loop. This problem is illustrated by the following example.

#### Example 1

**Fig. 1:**
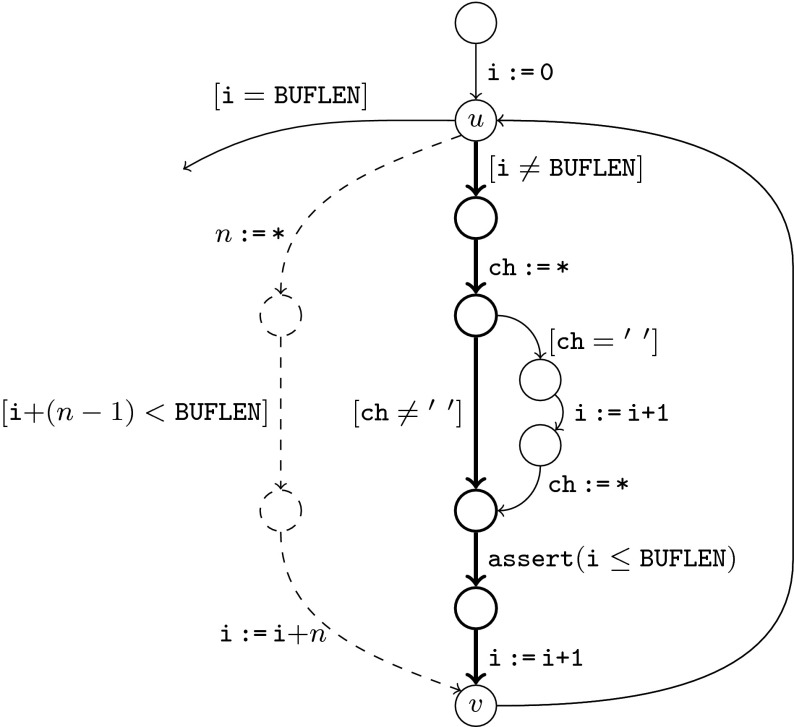
CFG with path $$\pi $$ (*bold*) and approximated path $$\mathop {\pi }\limits ^{\leadsto
												}$$ (*dashed*)

Figure 1 shows a program fragment derived from code permitting a buffer overflow (detected by the assertion) to occur in the $n$th iteration of the loop if $\mathtt{i}$ reaches $(\mathtt{BUFLEN}-1)$ and the branch $[\mathtt{ch}=\mathtt{'}~\mathtt{'}]$ is taken in the $(n-1)$th iteration. The verification techniques mentioned above explore the paths in order of increasing length. The shortest path that reaches the assertion does not violate it, as

$$\begin{aligned}
										sp({(\mathtt{i\,:=\,}0\mathtt{;}\,[\mathtt{i}\ne
										\mathtt{BUFLEN\}]\mathtt{;}\,
										\mathtt{ch\,:=\,*;}\,[\mathtt{ch}\ne
										\mathtt{'}~\mathtt{'}]})},{{\mathsf {T}}}) \quad \Rightarrow
										\quad (\mathtt{i}\le \mathtt{BUFLEN})\,.
										\end{aligned}$$

In a predicate abstraction or lazy abstraction framework, this path represents the first in a series of spurious counterexamples of increasing length. Let $$\pi $$ denote the path emphasised in Fig. 1, which traverses the loop once. The verification tool will generate a family of spurious counterexamples with the prefixes i := 0; $$\pi ^n$$ (where $$0<n\le
										\frac{\mathtt{BUFLEN}}{2}$$) before it detects a path long enough to violate the assertion. Each of these paths triggers a computationally expensive refinement cycle. Similarly, a bounded model checker will fail to detect a counterexample unless the loop bound is increased to $$\frac{\mathtt{BUFLEN}}{2}+1$$.

The iterative exploration of increasingly deeper loops primarily delays the detection of assertion violations (c.f. [16]), but can also result in a diverging series of interpolants and predicates if the program is safe (see [12]).

### Approximating paths with loops

We propose a technique that aims at avoiding the enumeration of paths with an insufficient number of loop iterations. Our approach is based on the insight that the refutation of spurious counterexamples containing a sub-path of the form $\pi ^n$ is futile if there exists an $n$ large enough to permit an assertion violation. We add an auxiliary path that bypasses the original loop body and represents the effect of $\pi ^n$ for a range of $n$ (detailed later in the paper). Our approach comprises the following steps:

1. We sensitise an existing tool to detect paths $$\pi $$ that repeatedly traverse the loop body B (as illustrated in the left half of Fig. 2). We emphasise that $$\pi $$ may span more than one iteration of the loop, and that the branches of B taken by $$\pi $$ in different iterations may vary.
2. We construct a path $$\mathop {\pi }\limits ^{\leadsto
												}$$ whose behaviour *under-approximates*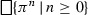. This construction does not correspond to acceleration in a strict sense, since $$\mathop {\pi }\limits ^{\leadsto
												}$$ (as an under-approximation) does not necessarily represent an arbitrary number of loop iterations. Section 3 describes techniques to derive $$\mathop {\pi }\limits ^{\leadsto
												}$$.
3. By construction, the assumptions in $$\mathop {\pi }\limits ^{\leadsto
												}$$ may contain universal quantifiers ranging over an auxiliary variable which encodes the number of loop iterations. In Sect. 4, we discuss two cases in which (some of) these quantifiers can be eliminated, namely (a) if the characteristic function of the predicate $$\lnot wlp\left( {\pi ^n},{\mathsf
												{F}}\right) $$ is *monotonic* in the number of loop iterations $$n$$, or (b) if $$\pi ^n$$ modifies an array and the indices of the modified array elements can be characterised by means of a quantifier-free predicate. We show that in certain cases condition (a) can be met by splitting $$\pi $$ into several separate paths.
4. We augment the control flow graph with an additional branch of the loop containing $$\mathop {\pi }\limits ^{\leadsto
												}$$ (Fig. 2, right). Section 5 demonstrates empirically how this program transformation can accelerate the detection of bugs that require a large number of loop iterations.

The following example demonstrates how our technique accelerates the detection of the buffer overflow of Example 1.

**Fig. 2:**
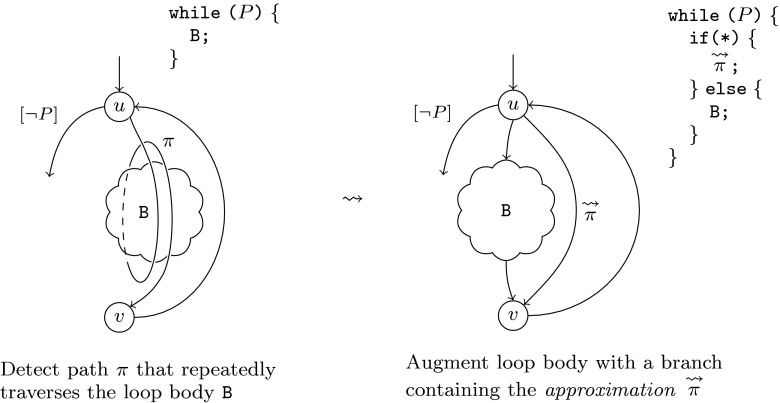
Approximating the natural loop with head $$u$$ and back-edge $$v\rightarrow
												u$$. Path $$\pi $$ is a path traversing the body B at least once, and may take different branches in B in subsequent iterations

#### Example 2

Assume that the verification tool encounters the node $$u$$ in Fig. 1 a second time during the exploration of a path ($$u$$ is the head of a natural loop with back-edge $$v\rightarrow u$$). We conclude that there exists a family of (sub-)paths $$\pi ^n$$ induced by the number $$n$$ of loop iterations. The repeated application of the strongest post-condition to the parametrised path $$\pi ^n$$ for an increasing $$n$$ gives rise to a recurrence equation $$\mathtt{i}^{\langle {n}\rangle
										}=\mathtt{i}^{\langle {n-1}\rangle
										}+1$$ (for clarity, we work on a sliced path omitting statements referring to ch):

$$\begin{aligned} \begin{array}{lll} sp({\pi
										^1},{{\mathsf {T}}}) &{}=&{} \exists
										\mathtt{i}^{\langle {0}\rangle }\,.\,(\mathtt{i}^{\langle
										{0}\rangle }<\mathtt{BUFLEN})\wedge
										(\mathtt{i}=\mathtt{i}^{\langle {0}\rangle }+1)\\ sp({\pi
										^2},{{\mathsf {T}}}) &{}=&{} \begin{aligned}
										\exists \mathtt{i}^{\langle {0}\rangle },\mathtt{i}^{\langle
										{1}\rangle }\,.\,&{} (\mathtt{i}^{\langle {0}\rangle
										}<\mathtt{BUFLEN}) \wedge (\mathtt{i}^{\langle
										{1}\rangle }<\mathtt{BUFLEN})\wedge \\ &{}
										(\mathtt{i}^{\langle {1}\rangle }=\mathtt{i}^{\langle
										{0}\rangle }+1)\wedge (\mathtt{i}=\mathtt{i}^{\langle
										{1}\rangle }+1) \end{aligned}\\ &{}\vdots
										&{}\\ sp({\pi ^n},{{\mathsf {T}}})
										&{}=&{} \exists \mathtt{i}^{\langle
										{0}\rangle }\ldots \mathtt{i}^{\langle {n-1}\rangle }\,.\,
										\left( \bigwedge _{j=0}^{n-1} (\mathtt{i}^{\langle
										{j}\rangle }<\mathtt{BUFLEN})\wedge
										(\mathtt{i}^{\langle {j+1}\rangle }= \mathtt{i}^{\langle
										{j}\rangle }+1)\right) \\ \end{array}
										\end{aligned}$$

where $$\mathtt{i}^{\langle {n}\rangle
										}$$ in the last line represents $$\mathtt{i}$$ after the execution of $$\pi ^n$$. This recurrence equation can be put into its equivalent closed form $$\mathtt{i}^{\langle {n}\rangle
										}=\mathtt{i}^{\langle {0}\rangle
										}+n$$. By assigning $$n$$ a (positive) non-deterministic value, we obtain the approximation (which happens to be exact in this case):

$$\begin{aligned} \mathop {\pi }\limits
										^{\leadsto }\quad =\quad n\,\mathtt{:=*;}\,[\forall j\in
										[0,n)\,.\,\mathtt{i}+j
										<\mathtt{BUFLEN}]\mathtt{;}\,\mathtt{i:=i}+n.
										\end{aligned}$$

Let us ignore arithmetic over- or under-flow for the time being (this topic is addressed in Sect. 3.4). We can then observe the following: if the predicate $$\mathtt{i}+j<\mathtt{BUFLEN}$$ is true for $$j=n-1$$, then it must be true for any $$j<n-1$$, i.e., the characteristic function of the predicate is *monotonic* in its parameter $$j$$. It is therefore possible to eliminate the universal quantifier and replace the assumption in $$\mathop {\pi }\limits ^{\leadsto
										}$$ with $$(\mathtt{i}+(n-1)<\mathtt{BUFLEN})$$. The dashed path in Fig. 1 illustrates the corresponding modification of the original program. The resulting transformed program permits the violation of the assertion in the original loop body after a single iteration of $$\mathop {\pi }\limits ^{\leadsto
										}$$ (corresponding to BUFLEN-1 iterations of $$\pi $$).

The following presents techniques to compute the under-approximation $$\mathop {\pi }\limits ^{\leadsto
										}$$.

## Under-approximation techniques

This section covers techniques to compute under-approximations $$\mathop {\pi
								}\limits ^{\leadsto }$$ of 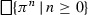 such that $$\mathop {\pi
								}\limits ^{\leadsto }$$ is a condensation of the CFG fragment to the right. Formally, we only require that $$ sp({\mathop
								{\pi }\limits ^{\leadsto }},{P}) \Rightarrow \exists n\in \mathbb
								{N}\,.\, sp({\pi ^n},{P}) $$ for all $$P$$.



The construction of $$\mathop {\pi
								}\limits ^{\leadsto }$$ has two aspects. Firstly, we need to make sure that all variables modified in $$\mathop {\pi
								}\limits ^{\leadsto }$$ are assigned values consistent with $$\pi
								^n$$ for a non-deterministic choice of $$n$$. Secondly, $$\mathop {\pi
								}\limits ^{\leadsto }$$ must only allow choices of $$n$$ for which $$\lnot wlp\left(
								{\pi ^n},{\mathsf {F}}\right) $$ is satisfiable, i.e., the corresponding path $$\pi
								^n$$ must be *feasible*.

Our approximation technique is based on the observation that the sequence of assignments in $$\pi
								^n$$ to a variable $$\mathtt{x}\in
								\mathtt{X}$$ corresponds to a recurrence equation (c.f. Example 2). The goal is to derive an equivalent *closed* form $$\mathtt{x\mathtt
								:=}\,f_\mathtt{x}(\mathtt{X}, n)$$. While there is a range of techniques to solve recurrence equations, we argue that it is sufficient to consider closed-form solutions that have the form of low-degree polynomials. The underlying argument is that a super-polynomial growth of variable values typically leads to an arithmetic overflow after a small number of iterations, which can be detected at low depth using conventional techniques.

The following sub-section focuses on deriving closed forms from a sequence of assignments to scalar integer variables, leaving conditionals aside. Section 3.2 covers assignments to arrays. Conditionals and path feasibility are addressed in Sect. 3.3. Section 3.4 addresses bit-vector semantics and arithmetic overflow.

### Computing closed forms of assignments

#### Syntactic matching

A simple technique to derive closed forms is to check whether the given recurrence equation matches a pre-determined format. In our work on loop detection for predicate abstraction [16, 17], we apply the following scheme:

1 $$\begin{aligned} \mathtt{x}^{\langle
										{0}\rangle } = \alpha ,\quad \mathtt{x}^{\langle {n}\rangle
										} = \mathtt{x}^{\langle {n-1}\rangle }+\beta +\gamma \cdot n
										\quad \leadsto \quad \mathtt{x}^{\langle {n}\rangle }=\alpha
										+\beta n+\gamma \frac{n\cdot (n+1)}{2}\,,
										\end{aligned}$$

where $$n>0$$ and $$\alpha $$, $$\beta $$, and $$\gamma $$ are numeric constants or loop-invariant *symbolic* expressions and x is the variant. This technique is computationally cheap and sufficient to construct the closed form $$\mathtt{i}^{\langle {n}\rangle
										}=\mathtt{i}^{\langle {0}\rangle
										}+n$$ of the recurrence equation $$\mathtt{i}^{\langle {n}\rangle
										}=\mathtt{i}^{\langle {n-1}\rangle
										}+1$$ derived from the assignment $$\mathtt{i:=
										i}+1$$ in Example 2.

#### Constraint-based acceleration

The disadvantage of a syntax-based approach is that it is limited to assignments following a simple pattern. Moreover, the technique is contingent on the syntax of the program fragment and may therefore fail even if there *exists* an appropriate polynomial representing the given assignments. In this section, we present an alternative technique that relies on a constraint solver to identify the coefficients of the polynomial $f_\mathtt{x}$.

Let $$\mathtt{X}$$ be the set $$\{\mathtt{x}_1,\ldots
										,\mathtt{x}_k\}$$ of variables in $$\pi $$. (In the following, we omit the braces $$\{\}$$ if clear from the context.) As previously, we start with the assumption that for each variable x modified in $$\pi $$, there is a low-degree polynomial in $$n$$

2 $$\begin{aligned}
										f_\mathtt{x}(\mathtt{X}^{\langle {0}\rangle }, n)\mathop
										{=}\limits ^{\mathrm{\tiny def}}\sum _{i=1}^k\alpha _i\cdot
										\mathtt{x}^{\langle {0}\rangle }_i + \left( \sum
										_{i=1}^k\alpha _{(k+i)}\cdot \mathtt{x}^{\langle {0}\rangle
										}_i + \alpha _{(2\cdot k+1)}\right) \cdot n + \alpha
										_{(2\cdot k +2)} \cdot n^2
										\end{aligned}$$

over the initial variables $$\mathtt{x}^{\langle {0}\rangle }_1,\ldots
										,\mathtt{x}^{\langle {0}\rangle
										}_k$$ which accurately represents the value assigned to x in $$\pi ^n$$ (for $$n\ge 1$$). In other words, for each variable $$\mathtt{x}\in
										\mathtt{X}$$ modified in $$\pi $$, we assume that the following Hoare triple is valid:

3 $$\begin{aligned} \left\{ { \bigwedge
										_{i=1}^k\,{}^{\backprime }\mathtt{x}_i = \mathtt{x}_i
										}\right\} \;{ \pi ^n }\;\left\{ { \mathtt{x} =
										f_\mathtt{x}({}^{\backprime }\mathtt{x}_1,\ldots
										,{}^{\backprime }\mathtt{x}_k, n)}\right\}
										\end{aligned}$$

For each $$\mathtt{x}\in \{\mathtt{x}_1,\ldots
										,\mathtt{x}_k\}$$ we can generate $$2\cdot k+2$$ distinct assignments to $${\mathtt{x}}^{\langle {0}\rangle }_1,
										\ldots , {\mathtt{x}}^{\langle {0}\rangle
										}_k$$, and $$n$$ in (2) which determine a system of linearly independent equations over $$\alpha _i,$$$$0< i\le 2\cdot
										k+2$$. If a solution to this system of equations exists, it *uniquely* determines the parameters $$\alpha _1,\ldots ,\alpha _{2\cdot
										k+2}$$ of the polynomial $$f_\mathtt{x}$$ for x. We will now examine the details of this construction and prove that it allows us to generate polynomial closed forms.

##### Lemma 1

A set of vectors $${\mathcal {X}} = \{\mathbf {x}_1,
											\ldots , \mathbf {x}_n \}$$ in vector space $$\mathcal
											{V}$$ is linearly independent if a projection of $${\mathcal
											{X}}$$ onto a subspace $${\mathcal
											{W}}$$ is linearly independent.

##### Proof

Let $${\mathcal {Y}} = \{ \mathbf {y}_1,
											\ldots , \mathbf {y}_m \} $$ be the projection of $${\mathcal
											{X}}$$ onto $${\mathcal
											{W}}$$. Assume for contradiction that $${\mathcal
											{X}}$$ is linearly dependent, then there is a set of scalars $$a_1, \ldots ,
											a_n$$ such that $$\sum _i a_i \mathbf {x}_i =
											\mathbf{0}$$. But when we project onto $${\mathcal
											{W}}$$ we have $$\sum _i a_i \mathbf {y}_i =
											\mathbf{0}$$, contradicting the assumption that $${\mathcal
											{Y}}$$ is linearly independent. $$\square $$

##### Theorem 1

We can uniquely determine the coefficients for a polynomial over $k$ variables by evaluating the loop at $2k+2$ points with $n \le 2$.

##### Proof

Our polynomial is of the form

$$\begin{aligned} \sum _{i=1}^k\alpha
											_i\cdot \mathtt{x}_i + \sum _{i=1}^k\alpha _{(k+i)}\cdot
											\mathtt{x}_i \cdot n + \alpha _{(2\cdot k+1)} \cdot n +
											\alpha _{(2\cdot k +2)} \cdot n^2
											\end{aligned}$$

There are $$2\cdot k+2$$ undetermined coefficients $$\alpha _i$$ ($$1\le i \le
											2k+2$$) that we need to find. We need to generate a system of $$2k+2$$ linearly independent equations to uniquely fix these coefficients. This is equivalent to finding a set of $$2k+2$$ vectors

$$\begin{aligned} (x_{(i,1)}, \ldots ,
											x_{(i,k)}, n_i)\quad \text {where}~1\le i\le 2\cdot k+2
											\end{aligned}$$

such that the set

$$\begin{aligned} \left\{ (x_{(i,1)},
											\ldots , x_{(i,k)}, x_{(i,1)}\cdot n, \ldots ,
											x_{(i,k)}\cdot n, n_i, n_i^2)\,\vert \, 1\le i\le 2\cdot
											k+1\right\} \end{aligned}$$

is linearly independent. We generate this set inductively.

*Basis* For $k=1$, the set generated by

$$\begin{aligned} \begin{array}{c}
											(x_{(1,1)}=1, n_1=0)\\ (0, 1)\\ (1, 1)\\ (1, 2)
											\end{array}\qquad \text {is}\qquad \begin{array}{c}
											(x_{(1,1)}=1, x_{(1,1)}\cdot n = 0, n_1 = 0, n_1^2 =
											0)\\ (0, 0, 1, 1)\\ (1, 1, 1, 1)\\ (1, 2, 2, 4)
											\end{array} \end{aligned}$$

which is linearly independent.

*Induction* Assume we have a linearly independent set of $2\cdot k+2$ equations for $k$ variables. We can extend the vectors by setting $x_{k+1}=0$ in the vectors with $n=0$ and $n=1$, and by setting $x_{k+1}=1$ in the vector with $n=2$. By Lemma 1 this maintains linear independence.

Subsequently, we add two new vectors generated by

$$\begin{aligned} \begin{array}{l}
											(n_{(2\cdot k+3)}=0, x_{(2\cdot k+3,k+1)}=1, x_{2\cdot
											k+3, j \ne k+1} = 0) \quad \text {and}\\ (n_{(2\cdot
											k+4)}=1, x_{(2\cdot k+4,k+1)}=1, x_{(2\cdot k+4,j \ne
											k+1)}=0)\,. \end{array}
											\end{aligned}$$

The resulting $$2\cdot k+4$$ vectors are still linearly independent, which can be seen by projecting onto the space $$(x_{k+1}, x_{k+1}\cdot n, n,
											n^2)$$—the only way to generate the $$\mathbf{0}$$ vector is by taking combinations of vectors all of which have $$x_{k+1}=0$$. But that set is a subset of the $$2\cdot k+2$$ equations we started with, which are linearly independent, and so the extended set is also linearly independent by Lemma 1. So we have $$2\cdot k+4 = 2\cdot (k+1) +
											2$$ linearly independent vectors and the induction is complete. $$\square $$

In particular, the satisfiability of the encoding from which we derive the assignments guarantees that (3) holds for $0\le n\le 2$. For larger values of $n$, we check the validity of (3) with respect to each $f_\mathtt{x}$ by means of induction. The validity of (3) follows (by induction over the length of the path $\pi ^n$) from the validity of the base case established above, the formula (4) given below (which can be easily checked using a model checker or a constraint solver), and Hoare’s rule of composition:

4 $$\begin{aligned} \left\{ {\bigwedge
											_{i=1}^k\left( {}^{\backprime }\mathtt{x}_i =
											\mathtt{x}_i\right) \wedge \mathtt{x} =
											f_\mathtt{x}({}^{\backprime }\mathtt{x}_1,\ldots
											,{}^{\backprime }\mathtt{x}_k, n)}\right\} \;{ \pi
											}\;\left\{ { \mathtt{x} = f_\mathtt{x}({}^{\backprime
											}\mathtt{x}_1,\ldots ,{}^{\backprime }\mathtt{x}_k,
											n+1)}\right\} \end{aligned}$$

If for one or more $$\mathtt{x}\in \{\mathtt{x}_1,\ldots
											,\mathtt{x}_k\}$$ our technique fails to find valid parameters $$\alpha _1,\ldots ,\alpha _{2\cdot
											k+2}$$, or the validity check for $$f_\mathtt{x}$$ fails, we do not construct $$\mathop {\pi }\limits ^{\leadsto
											}$$.

##### Remark

The construction of under-approximations is not limited to the two techniques discussed above and can be based on other (and potentially more powerful) recurrence solvers. that are commonly applied in compiler construction [23] and in the context of invariant generation (see [14], for instance). While techniques such as [7] support a larger class of recurrence equations, our approach suffices cover the most common cases like linear counters. Given that we restrict acceleration to intervals in which overflows can be ruled out (see Sect. 3.4), there is no necessity to handle loop counters that increase exponentially, as these cases can be handled efficiently by traditional unwinding.

### Assignments to arrays

Buffer overflows constitute a prominent class of safety violations that require a large number of loop iterations to surface. In C programs, buffers and strings are typically implemented using arrays. Let i be the variant of a loop which contains an assignment a[i]:=$e$ to an array a. For a single iteration, we obtain

5 $$\begin{aligned} sp({\mathtt{a[i]:=}\,e},{P})
									\mathop {=}\limits ^{\mathrm{\tiny def}}\exists {}^{\backprime
									}\mathtt{a}\,.\,\mathtt{a[i]}=e[\mathtt{a}/{}^{\backprime
									}\mathtt{a}]\wedge \forall j\ne \mathtt{i}\,.\left(
									\mathtt{a}[j]=\mathtt{{}^{\backprime }{a}}[j]\right) \wedge
									P[\mathtt{a}/{}^{\backprime }\mathtt{a}]
									\end{aligned}$$

Assume further that closed forms for the variant i and the expression $e$ exist (abusing our notation, we use $f_e$ to refer to the latter). Given an initial pre-condition $P={\mathsf {T}}$, we obtain the following *under-approximation* after $n$ iterations:

6 $$\begin{aligned}&\forall j\in
									[0,n)\,.{\mathtt{a}}^{\langle {n}\rangle
									}[f_\mathtt{i}(\mathtt{X}^{\langle {0}\rangle },j)]=
									f_e(\mathtt{X}^{\langle {0}\rangle },j)\;\wedge \nonumber
									\\&\quad \forall i\in {{\mathrm{dom}}}\mathtt{a}\,.\,
									\underbrace{\left( \exists j\in [0,n)\,.\,
									i=f_\mathtt{i}(\mathtt{X}^{\langle {0}\rangle }, j)\right) }_{
									\text {membership test}} \;\vee \; \left( \mathtt{a}^{\langle
									{n}\rangle }[i]={\mathtt{a}}^{\langle {0}\rangle }[i]\right) \,,
									\end{aligned}$$

where the domain $$({{\mathrm{dom}}}\mathtt{a})$$ of a denotes the valid indices of the array. Condition (6) *under-*approximates the strongest post-condition, since there may exist $$j_1,j_2\in [0,n)$$ such that $$j_1\ne j_2\wedge
									f_\mathtt{i}(\mathtt{X}^{\langle {0}\rangle
									},j_1)=f_\mathtt{i}(\mathtt{X}^{\langle {0}\rangle
									},j_2)$$ and (6) is unsatisfiable. A similar situation arises if a loop body $$\pi $$ contains multiple updates of the same array.

Notably, the membership test determining whether an array element is modified or not introduces quantifier alternation, posing a challenge to contemporary decision procedures. Section 4 addresses the elimination of the existential quantifier.

### Assumptions and feasibility of paths

The techniques discussed in Sect. 3.1 yield polynomials and constraints representing the assignment statements of $$\pi ^n$$, but leave aside the conditional statements which determine the feasibility of the path. In order to guarantee that only states that are reachable in the original program can be reached via accelerated paths, we need to make sure that $$\mathop {\pi }\limits ^{\leadsto
									}$$ is only feasible for values of $$n$$ for which $$\pi ^n$$ is also feasible. We achieve this by computing a pre-condition for $$\mathop {\pi }\limits ^{\leadsto
									}$$ that rules out values of $$n$$ for which $$\pi ^n$$ is not feasible. In the following, we demonstrate how to derive such a pre-condition $$\lnot wlp\left( {\pi ^n},{\mathsf {F}}\right)
									$$ using the polynomials $$f_\mathtt{x}$$ for $$\mathtt{x}\in
									\mathtt{X}$$.

Let $$f_\mathtt{X}(\mathtt{X}, n)\mathop {=}\limits
									^{\mathrm{\tiny def}}\{f_\mathtt{x}(\mathtt{X},n)\,\vert
									\,\mathtt{x}\in \mathtt{X}\}$$ and let $$Q[\mathtt{X}/f_\mathtt{X}(\mathtt{X},n)]$$ denote the simultaneous substitution of all free occurrences of the variables $$\mathtt{x}\in
									\mathtt{X}$$ in $$Q$$ with the corresponding term $$f_\mathtt{x}(\mathtt{X},
									n)$$. Accordingly, given a path $$\pi $$ modifying the set of variables X and a corresponding set $$f_\mathtt{X}$$ of closed-form assignments, we can construct an accurate representation of $$\pi ^n$$ as follows:

7 $$\begin{aligned} \underbrace{ [\,\forall j\in
									[0,n)\,.\left( \lnot wlp\left( {\pi },{\mathsf {F}}\right)
									\right) [\mathtt{X}/f_\mathtt{X}(\mathtt{X}, j)]\,]}_{\text
									{satisfiable if }\pi ^n\text { is feasible}} \quad
									\mathtt{;}\quad
									\underbrace{\mathtt{X\,:=}f_\mathtt{X}(\mathtt{X},n)}_{\text
									{assignments of }\pi ^n}
									\end{aligned}$$

The construction of $$\lnot wlp\left( {\pi ^n},{\mathsf {F}}\right)
									$$ is based on the following lemma:

#### Lemma 2

The following equivalence holds:

$$\begin{aligned} wlp\left( {\pi
										^n},{\mathsf {F}}\right) \equiv \exists j\in [0,n)\,.\left(
										wlp\left( {\pi },{\mathsf {F}}\right) \right)
										[\mathtt{X}/f_\mathtt{X}(\mathtt{X},j)]
										\end{aligned}$$

#### Proof

Intuitively, the path $$\pi ^n$$ is infeasible if for *any*$$j<n$$ the first time-frame of the suffix $$\pi ^{(n-j)}$$ is infeasible. We prove the claim by induction over $$n$$. Due to (3) and (4) we have $$f_\mathtt{X}(\mathtt{X},
										0)=\mathtt{X}$$ and $$f_\mathtt{X}(f_\mathtt{X}(\mathtt{X}, n),
										1)=f_\mathtt{X}(\mathtt{X}, n+1)$$ (for $$n\ge 0$$).

*Base case*$$ wlp\left( {\pi },{\mathsf {F}}\right)
										\equiv \exists j\in [0,0)\,.\left( wlp\left( {\pi },{\mathsf
										{F}}\right) \right)
										[\mathtt{X}/f_\mathtt{X}(\mathtt{X},j)]=\mathsf
										{F}$$

*Induction step* We start by applying the induction hypothesis:

$$\begin{aligned} \begin{array}{rcl}
										wlp\left( {\pi ^n},{\mathsf {F}}\right) &{}\equiv
										&{} wlp\left( {\pi },{\left( wlp\left( {\pi
										^{n-1}},{\mathsf {F}}\right) \right) }\right) \\
										&{}\equiv &{} wlp\left( {\pi },{ \exists
										j\in [0,n-1)\,.\left( wlp\left( {\pi },{\mathsf {F}}\right)
										\right) [\mathtt{X}/f_\mathtt{X}(\mathtt{X},j)]}\right)
										\end{array} \end{aligned}$$

We consider the effect of assignments and assumptions occurring in $$\pi $$ on the post-condition $$Q\mathop {=}\limits ^{\mathrm{\tiny
										def}}\left( \exists j\in [0,n-1)\,.\left( wlp\left( {\pi
										},{\mathsf {F}}\right) \right)
										[\mathtt{X}/f_\mathtt{X}(\mathtt{X},j)]\right)
										$$ separately.

- The effect of assignments in $$\pi $$ on $$Q$$ is characterised by $$Q[\mathtt{X}/f_\mathtt{X}(\mathtt{X},1)]$$. We obtain:
  $$\begin{aligned}
												Q[\mathtt{X}/f_\mathtt{X}(\mathtt{X},1)]\;\equiv
												& {} \; \exists j\in [0,n-1)\,.\left(
												wlp\left( {\pi },{\mathsf {F}}\right) \right)
												[\mathtt{X}/f_\mathtt{X}(f_\mathtt{X}(\mathtt{X},
												1),j)]\;\\\equiv & {} \exists j\in
												[1,n)\,.\left( wlp\left( {\pi },{\mathsf
												{F}}\right) \right)
												[\mathtt{X}/f_\mathtt{X}(\mathtt{X},j)]\nonumber
												\end{aligned}$$
- Assumptions in $$\pi $$ contribute the disjunct $$ wlp\left( {\pi },{\mathsf
												{F}}\right) $$.

By combining both contributions into one term we obtain

$$\begin{aligned} wlp\left( {\pi
										^n},{\mathsf {F}}\right) \equiv \left( wlp\left( {\pi
										},{\mathsf {F}}\right) \right)
										[\mathtt{X}/f_\mathtt{X}(\mathtt{X},0)]\vee \exists j\in
										[1,n)\,.\left( wlp\left( {\pi },{\mathsf {F}}\right) \right)
										[\mathtt{X}/f_\mathtt{X}(\mathtt{X},j)]\;,
										\end{aligned}$$

which establishes the claim of Lemma 2. $\square$

We emphasise that our construction (unlike many acceleration techniques such as [11]) does *not* restrict the assumptions in $$\pi $$ to a limited class of relations on integers. The construction of the path (7), however, does require closed forms of all assignments in $$\pi $$. Since we do not construct closed forms for array assignments (as opposed to assignments to array indices, c.f. Sect. 3.2), we cannot apply Lemma 2 if $$ wlp\left( {\pi },{\mathsf {F}}\right)
										$$ refers to an array assigned in $$\pi $$. In this case, we do not construct $$\mathop {\pi }\limits ^{\leadsto
										}$$.

For assignments of variables not occurring in $$ wlp\left( {\pi },{\mathsf {F}}\right)
										$$, we augment the domain(s) of the variables X with an undefined value $$\bot $$ (implemented using a Boolean flag) and replace $$f_\mathtt{x}$$ with $$\bot $$ whenever the respective closed form is not available. Subsequently, whenever the search algorithm encounters an (abstract) counterexample, we use slicing to determine whether the feasibility of the counterexample depends on an undefined value $$\bot $$. If this is the case, the counterexample needs to be dismissed. Thus, any path $$\mathop {\pi }\limits ^{\leadsto
										}$$ containing references to $$\bot $$ is an *under-approximation* of $$\pi ^n$$ rather than an acceleration of $$\pi $$.

#### Example 3

For a path $$\pi \mathop {=}\limits ^{\mathrm{\tiny
										def}}[\mathtt{x}<10];\,\mathtt{x:=x+1;}\,\mathtt{y:=y^2}$$, we obtain the under-approximation $$\mathop {\pi }\limits ^{\leadsto }\equiv
										n\mathtt{:=*;}\,[\forall j\in [0,n).
										\mathtt{x}+j<10]\mathtt{;}\,
										\mathtt{x:=x}+n\mathtt{;}\,\mathtt{y:=}\bot
										$$. A counterexample traversing $$\mathop {\pi }\limits ^{\leadsto
										}$$ is feasible if its conditions do not depend on y.

### Arithmetic overflows

The fact that the techniques in Sect. 3.1 used to derive closed forms do not take arithmetic overflow into account may lead to undesired effects. For instance, the assumption made in Example 2 that the characteristic function of the predicate $$(\mathtt{i}+n<\mathtt{BUFLEN})$$ is monotonic in $$n$$ does not hold in the context of bit-vectors or modular arithmetic. Since, moreover, the behaviour of arithmetic over- or under-flow in C is not specified in certain cases, we conservatively rule out all occurrences thereof in $$\mathop {\pi }\limits ^{\leadsto
									}$$. For the simple assignment $$\mathtt{i\,:=\,i}+n$$ in Example 2, this can be achieved by adding the assumption $$(\mathtt{i}+n\le
									2^l-1)$$ to $$\mathop {\pi }\limits ^{\leadsto
									}$$ (for unsigned $$l$$-bit vectors). In general, we have to add respective assumptions $$(e_1\otimes e_2 \le
									2^l-1)$$ for all arithmetic (sub-)expressions $$e_1\otimes e_2$$ of bit-width $$l$$ and operations $$\otimes $$ in $$\mathop {\pi }\limits ^{\leadsto
									}$$.

While this approach is *sound* (eliminating paths from $$\mathop {\pi }\limits ^{\leadsto
									}$$ does not affect the correctness of the instrumented program, since all behaviours following an overflow are still reachable via non-approximated paths), it imposes restrictions on the range of $$n$$. Therefore, the resulting approximation $$\mathop {\pi }\limits ^{\leadsto
									}$$ deviates from the acceleration $$\pi ^*$$ of $$\pi $$. Unlike acceleration over linear affine relations, this adjustment makes our approach bit-level accurate. We emphasise that the benefit of the instrumentation can still be substantial, since the number of iterations required to trigger an arithmetic overflow is typically large.

### Path selection

In the following, we discuss heuristics to select paths $\pi$ to accelerate. Depending on which model checking technique we are incorporating acceleration into, several path selection strategies are available. Some model checkers already come equipped with a strategy for enumerating paths, for example Impact [19] enumerates paths by iteratively unrolling the CFG of the program under analysis. During this process, if a path is found to be “looping” (i.e. some program location is visited repeatedly) then that path is a candidate for acceleration. By contrast, if we use a model check technique that is not explicitly path based (such as bounded model checking), we must devise a strategy for selecting paths to accelerate.

A necessary condition for $\pi$ to be acceleratable is that $\pi ^2$ is feasible, for if it were not, $\pi ^n$ (for $n > 1$) would be infeasible, resulting in a trivial accelerator. Accordingly, paths $\pi$ for which $\pi ^2$ is feasible are promising candidates for acceleration. We can find such paths by using symbolic execution to build a system of constraints and solving the system with a SAT solver. Our encoding guarantees that if these constraints have a solution, the solution includes a path $\pi$ where $\pi ^2$ is feasible. Our encoding can be easily generalised and applied to paths $\pi ^k$ with a higher number $k$ of iterations, though this results in a higher computational effort. In practice, choosing $k=2$ results in a reliable predictor that enables us to identify candidates efficiently.

Let $$L$$ denote the program fragment denoting the loop body. We instrument $$L$$ with “distinguisher” and “shadow distinguisher” variables, which indicate which branches are taken. Concretely, for each statement 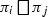 in $$L$$ we create boolean variables $$d_i$$, $$s_i$$, $$d_j$$, and $$s_j$$. We create the instrumented program $$\text {Instr}(L)$$ by prepending the statement $$d_i \mathtt{:=
									false}$$, appending the statement $$\mathtt{assume}(d_i =
									s_i)$$, and then replacing the statement 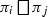 with
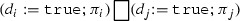
$$\text {Instr}(L)$$ has the property that when it has finished executing, each of the $$d_i$$ will be true iff the corresponding branch was taken. Furthermore, we have $$d_i = s_i$$ for each $$i$$. We now sequentially compose two copies of this instrumented program:

$$\begin{aligned} \text {Instr}(L) ; \text
									{Instr}(L) \end{aligned}$$

**Fig. 3:**
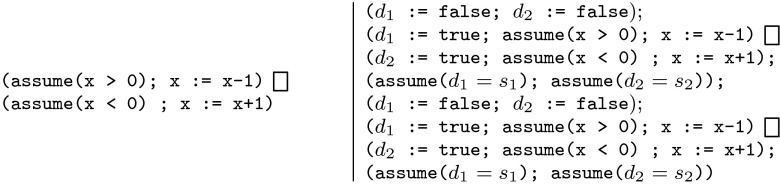
A program instrumented to enumerate acceleratable paths

We assume that the $s_i$ are initialised non-deterministically at the beginning of this program fragment. An example of this construction is shown in Fig. 3.

Since each of the distinguisher variables $$d_i$$ is equal to the shadow distinguisher $$s_i$$ at the end of each copy of $$\text {Instr}(L)$$, we know that the only feasible paths through this program are those in which both copies took the same path. This path is identified by the values of the $$s_i$$. So if there are *any* feasible paths through this program, they identify a path $$\pi $$ such that $$\pi ^2$$ in the original program is feasible. We can identify a feasible path through this program by appending the statement assert(false) to the end of the program and using a BMC-based model checker (which ultimately creates a SAT/SMT instance) to check the safety of the constructed program. We can iterate this process to enumerate candidate paths: if we have previously found the paths $$\pi _1, \ldots , \pi
									_n$$ we can add assumptions to the end of our path-enumerating program to prevent the rediscovery of these $$\pi _i$$.

## Eliminating quantifiers from approximations

A side effect of the approximation steps in Sects. 3.2 and 3.3 is the introduction of quantified assumptions. While quantification is often unavoidable in the presence of arrays, it is a detriment to performance of the decision procedures underlying the verification tools. In the worst case, quantifiers may result in the undecidability of path feasibility.

In the following, we discuss two techniques to eliminate or reduce the number of quantifiers in assumptions occurring in $$\mathop {\pi
								}\limits ^{\leadsto }$$.

### Eliminating quantifiers over monotonic predicates

We show that the quantifiers introduced by the technique presented in Sect. 3.3 can be eliminated if the predicate is monotonic in the quantified parameter.

#### Definition 1

(*Representing function, monotonicity*) The representing function $$f_P$$ of a predicate $$P$$ with the same domain takes, for each domain value, the value 0 if the predicate holds, and 1 if the predicate evaluates to false, i.e., $$P(\mathtt{X})\Leftrightarrow
										f_P(\mathtt{x})=0$$. A predicate $$P(n): {\mathbb {N}}\rightarrow {\mathbb
										{B}}$$ is monotonically increasing (decreasing) if its representing function $$f_P(n):{\mathbb {N}}\rightarrow {\mathbb
										{N}}$$ is monotonically increasing (decreasing), i.e., $$\forall m,n\,.\,m\le n\Rightarrow
										f_P(m)\le f_P(n)$$.

We extend this definition to predicates over variables X and $$n\in {\mathbb
										{N}}$$ as follows: $$P(\mathtt{X},n)$$ is monotonically increasing in $$n$$ if $$(m\le n)\wedge P(\mathtt{X}, n)\wedge
										\lnot P(\mathtt{X}, m)$$ is unsatisfiable.

#### Proposition 1

$$P(\mathtt{X},n-1)\equiv \forall i\in
										[0,n)\,.\, P(\mathtt{X},i)$$ if $$P$$ is monotonically increasing in $$i$$.

The validity of Proposition 1 follows immediately from the definition of monotonicity. Accordingly, it is legitimate to replace universally quantified predicates in $$\mathop {\pi }\limits ^{\leadsto
										}$$ with their corresponding unquantified counterparts (c.f. Proposition 1).

This technique, however, fails for simple cases such as $$\mathtt{x}\ne c$$ ($$c$$ being a constant). In certain cases, the approach can still be applied after *splitting* a non-monotonic predicate $$P$$ into monotonic predicates $$\{P_1,\ldots
										,P_m\}$$ such that $$P\equiv \bigvee _{i=1}^m
										P_i$$ (as illustrated in the Figure to the right). Subsequently, the path $$\pi $$ guarded by $$P$$ can be split as outlined in Fig. 4. This transformation preserves reachability (a proof for $$m=2$$ is given in Fig. 4).

**Fig. 4:**
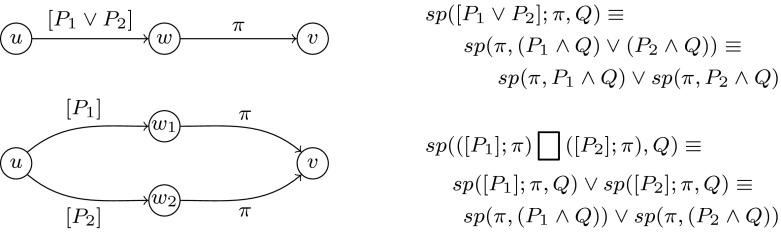
Splitting disjunctive assumptions preserves program behaviour

This approach is akin to trace partitioning [9], however, our intent is quantifier elimination rather than refining an abstract domain. We rely on a template-based approach to identify predicates that can be split (a constraint solver-based approach is bound to fail if $c$ is symbolic). While this technique effectively deals with a broad number of standard cases, it does fail for quantifiers over array indices, since the array access operation is not monotonic.



### Eliminating quantifiers in membership tests for array indices

This sub-section aims at replacing the existentially quantified membership test in Predicate (6) by a quantifier-free predicate. To define a set of sufficient (but not necessary) conditions for when this is possible, we introduce the notion of increasing and dense array indices (c.f. [13]):

#### Definition 2

*(Increasing and Dense Variables)* A scalar variable x is (strictly) *increasing* in $$\pi ^n$$ iff $$\forall j\in [0,n)\,.\,\mathtt{x}^{\langle
										{j+1}\rangle }\ge \mathtt{x}^{\langle {j}\rangle
										}$$ ($$\forall j\in [0,n)\,.\,\mathtt{x}^{\langle
										{j+1}\rangle }> \mathtt{x}^{\langle {j}\rangle
										}$$, respectively). Moreover, an increasing variable i is *dense* iff

$$\begin{aligned} \forall j\in
										[0,n)\,.\,\left( \mathtt{x}^{\langle {j+1}\rangle
										}=\mathtt{x}^{\langle {j}\rangle }\right) \vee \left(
										\mathtt{x}^{\langle {j+1}\rangle }=\mathtt{x}^{\langle
										{j}\rangle }+1\right) \,.
										\end{aligned}$$

Variables decreasing in $\pi ^n$ are defined analogously. A variable is monotonic (in $\pi ^n$) if it is increasing or decreasing (in $\pi ^n$).

Note that if the closed form $$f_\mathtt{x}(\mathtt{X}^{\langle
										{0}\rangle },n)$$ of a variable x is a linear polynomial, then x is necessarily monotonic. The following proposition uses this property:

#### Proposition 2

Let $$f_\mathtt{x}(\mathtt{X}^{\langle
										{0}\rangle },j)$$ be the closed form (2) of $$\mathtt{x}^{\langle {j}\rangle
										}$$, where $$\alpha _{(2\cdot k
										+2)}=0$$, i.e., the polynomial $$f_\mathtt{x}$$ is linear. Then $$\Delta f_\mathtt{x} \mathop {=}\limits
										^{\mathrm{\tiny def}}f_\mathtt{x}(\mathtt{X}^{\langle
										{0}\rangle },j+1)-f_\mathtt{x}(\mathtt{X}^{\langle
										{0}\rangle },j)$$ (for $$j\in [0,n)$$) is the (symbolic) constant $$\sum _{i=1}^k\alpha _{(k+i)}\cdot
										\mathtt{x}^{\langle {0}\rangle }_i+\alpha _{(2\cdot
										k+1)}$$. The variable x is (strictly) increasing in $$\pi ^n$$ if $$\Delta f_\mathtt{x}\ge
										0$$ ($$\Delta
										f_\mathtt{x}>0$$, respectively) and dense if $$0\le \Delta f_\mathtt{x}\le
										1$$.

#### Lemma 3

Let $$f_\mathtt{x}(\mathtt{X}^{\langle
										{0}\rangle },j)$$ be a linear polynomial representing the closed form (2) of $$\mathtt{x}^{\langle {j}\rangle
										}$$ (as in Proposition 2). The following logical equivalence holds:

8 $$\begin{aligned}&\exists j\in
										[0,n)\,.\, \mathtt{x}=f_\mathtt{x}(\mathtt{X}^{\langle
										{0}\rangle }, j)\;\equiv \;\nonumber \\&\quad
										\left\{ \begin{array}{ll} ((\mathtt{x}-\mathtt{x}^{\langle
										{0}\rangle })\mod \Delta f_\mathtt{x}=0)\wedge \left(
										\frac{\mathtt{x}-\mathtt{x}^{\langle {0}\rangle }}{\Delta
										f_\mathtt{x}}<n\right) &{} \quad \text {if }
										\mathtt{x} \text { is strictly increasing}\\
										\mathtt{x}-\mathtt{x}^{\langle {0}\rangle } \le (n-1)\cdot
										\Delta f_\mathtt{x} &{} \quad \text {if } \mathtt{x}
										\text { is dense}\\ \mathtt{x}-\mathtt{x}^{\langle
										{0}\rangle } < n &{} \quad \text {if both of
										the above hold}\\ \end{array}\right.
										\end{aligned}$$

The validity of Lemma 3 follows immediately from Proposition 2. Using Lemma 3, we can replace the existentially quantified membership test in Predicate (6) by a quantifier-free predicate if one of the side conditions in (8) holds. Given that the path prefix reaches the entry node of a loop, these conditions $$\Delta
										f_\mathtt{x}>0$$ and $$0\le \Delta f_\mathtt{x}\le
										1$$ can be checked using a satisfiability solver.

#### Example 4

Let $$\pi \mathop {=}\limits ^{\mathrm{\tiny
										def}}\mathtt{a[x]:=x;\,x:=x+1}$$ be the body of a loop. By instantiating (6), we obtain the condition

$$\begin{aligned} \forall j\in
										[0,n)\,.\mathtt{a}[{}^{\backprime }\mathtt{x}+j]=
										\mathtt{{}^{\backprime }{x}}+j\;\wedge \forall i. \left(
										\exists j\in [0,n)\,.\, i=\mathtt{{}^{\backprime
										}{x}}+j\right) \;\vee \; \left(
										\mathtt{a}[i]=\mathtt{{}^{\backprime }{a}}[i]\right) ,
										\end{aligned}$$

in which the existentially quantified term can be replaced by $$\mathtt{x}-\mathtt{{}^{\backprime
										}{x}}<n$$.

## Implementation and experimental results

Our under-approximation technique is designed to extend existing verifiers. To demonstrate its versatility, we implemented Impulse, a tool combining under-approximation with the two popular software verification techniques lazy abstraction with interpolants (LAwI) [19] and bounded model checking (specifically, Cbmc  [5]). The underlying SMT solver used throughout was version 4.2 of Z3. Impulse comprises two phases:

1. Impulse first explores the paths of the CFG following the LAwI paradigm. If Impulse encounters a path containing a loop with body $$\pi $$, it computes $$\mathop {\pi }\limits ^{\leadsto
											}$$ (processing inner loops first in the presence of nested loops), augments the CFG accordingly, and proceeds to phase 2.
2. Cbmc inspects the instrumented CFG up to an iteration bound of 2. If no counterexample is found, Impulse returns to phase 1.

In phase 1, spurious counterexamples serve as a catalyst to refine the current approximation of safely reachable states, relying on the weakest precondition^1^ to generate the required Hoare triples. Phase 2 takes advantage of the aggressive path merging performed by Cbmc, enabling fast counterexample detection.

We evaluated the effectiveness of under-approximation on the Verisec benchmark suite [18], which consists of manually sliced versions of several open source programs that contain buffer overflow vulnerabilities. We chose Verisec over the small synthetic benchmarks in the loop-acceleration category of the competition on software verification [2], since the Verisec suite is based on real-world vulnerabilities. 

**Fig. 5:**
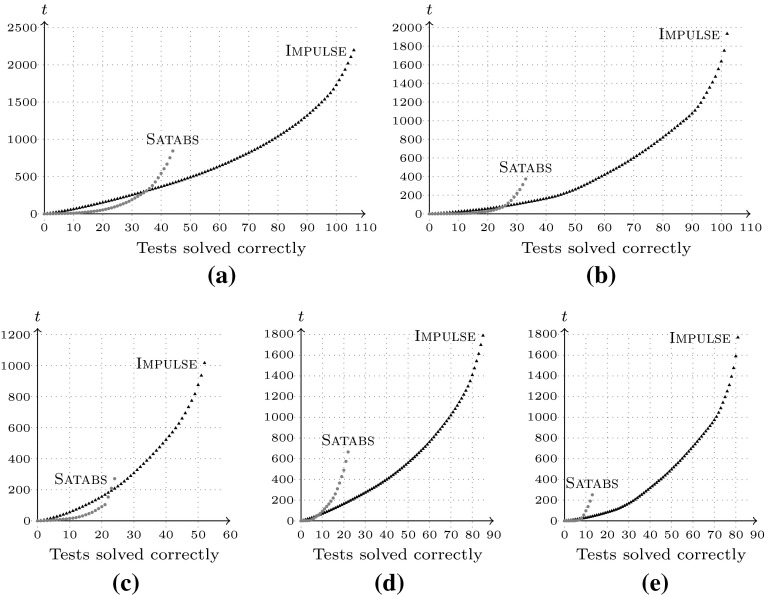
Verification run-times (cumulative) of Verisec benchmark suite, **a** safe and unsafe, buffer size 10, **b** safe and unsafe, buffer size $10^2$, **c** safe/unsafe, b.-size $10^3$, **d** unsafe, buffer size $10$, **e** unsafe, buffer size $10^2$

Of the 284 test cases of Verisec, 144 are labelled as containing a buffer overflow, and 140 are labelled as safe.^2^ The safety violations range from simple unchecked string copy into static buffers, up to complex loops with pointer arithmetic. The buffer size in each benchmark (c.f. BUFLEN in Fig. 1) is adjustable and controls the depth of the counterexample. We compared our tool with Satabs (which outperforms Impulse w/o approximation) on buffer sizes of 10, 100 and 1000, with a time limit of 300 s and a memory limit of 2 GB on an 8-core 3 GHz Xeon CPU. Figures 5a through 5c show the cumulative run-time for the whole benchmark suite, whereas Fig. 5d and e show only unsafe program instances. Table 2 provides an overview of the test cases solved by Impulse with or without acceleration compared to the test cases solved by Satabs, including the number of loops and programs that were accelerated. Further, our static acceleration tool accelerates loops (with symbolic rather than static bounds) in 42 programs out of the 79 candidates from the 2013 software verification competition.

**Table 2:** Number of accelerated loops (in 284 programs)

Tool	Solved	# Loops accelerated	# Programs accelerated
Impulse (w/o acc.)	17	0	0
Impulse	102	258	119
Satabs	33	0	0

Finally, on the 8 safe instances from the Verisec benchmark that Impulse can solve (using interpolants computed via the weakest pre-condition), under-approximation did not improve (or impair) the run-time on safe instances.

**Fig. 6:**
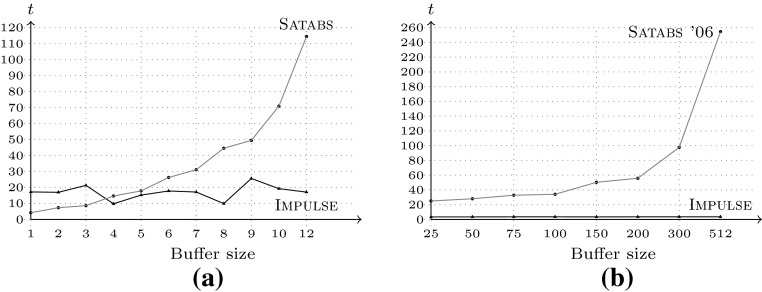
Run-time dependency on buffer size for unsafe benchmarks, **a** single Verisec test, varying buffer size, **b** Satabs w. loop detect on Aeon 0.02a

Figure 6 demonstrates that the time Impulse requires to detect a buffer overflow does not depend on the buffer size. Figure 6a compares Satabs and Impulse on a single Verisec benchmark with a varying size parameter, showing that Satabs takes time exponential in the size of the buffer. Figure 6b provides a qualitative comparison of the loop-detection technique presented in [16] with Impulse on the Aeon 0.02a mail transfer agent. Figure 6b shows the run-times of Satabs ’06 with loop detection as reported in [16],^3^ as well as the run-times of Impulse on the same problem instances and buffer sizes. Satabs ’06 outperforms similar model checking tools that do not feature loop-handling mechanisms [16]. However, the run-time still increases exponentially with the size of the buffer, since the technique necessitates a validation of the unwound counterexample. Impulse does not require such a validation step.

## Related work

The under-approximation technique presented in this paper is based on our previous work on loop detection [16, 17]. The algorithm in [16], however, does not yield a strict under-approximation, and thus necessitates an additional step to validate the unwound counterexample. Our new technique avoids this problem.

The techniques in Sect. 3.1 constitute a simple form of acceleration [4, 8]. The subsequent restrictions in Sects. 3.3 and 3.4 on $$\mathop {\pi
								}\limits ^{\leadsto }$$, however, impose a symbolic bound on the number of iterations, yielding an under-approximation. In contrast to acceleration of integer relations, our approximation is sound for bit-vector arithmetic. Sinha uses term rewriting to compute symbolic states parametrised by the loop counter, stating that his technique can be extended to support bit-vectors [22].

The quantifier elimination technique of Sect. 4.1 bears similarities with splitter predicates [21], a program transformation facilitating the generation of disjunctive invariants. Similarly, trace partitioning [9] splits program paths to increase the precision of static analyses. Neither technique aims at eliminating quantifiers.

Loop summarisation [15] and path invariants [3] avoid loop unrolling by selecting an appropriate over-approximation of the loop from a catalogue of invariant templates. Over-approximations are also used in the context of loop bound inference [24] and reasoning about termination [6]. However, over-approximations do not enable the efficient detection of counterexamples.

Hojjat et al. [11] uses interpolation to derive inductive invariants from accelerated paths. While this work combines under- and over-approximation, it is not aimed at counterexample detection. Motivated by the results of [11], we believe that under-approximation can, if combined with interpolation, improve the performance of verification tools on safe programs. We plan to support interpolation in a future version of our implementation.

Techniques to solve recurrences are frequently used in compiler construction [23]. For example, van Engelen et al. [7] present an algorithm to compute closed forms for systems of recurrence equations, which also supports induction variables that are updated conditionally by introducing a dynamic range for each variable. While it would be straight forward to incorporate these ideas into our work, the technique discussed in Sect. 3 is sufficient for all practical purposes if the bounded range of program variables is taken into account (see our remark at the end of Sect. 3.1.2).

We refer the reader to [17] for a description of additional related work.

## Conclusion and future work

We present a sound under-approximation technique for loops in C programs with bit-vector semantics. The approach is very effective for finding deep counterexamples in programs that manipulate arrays, and compatible with a variety of existing verification techniques. A short-coming of our under-approximation technique is its lack of support for dynamic data structures, which we see as a challenging future direction.

## Footnotes

1 In a preliminary interpolation-based implementation, Z3 was in many cases unable to provide interpolants for path formulas $$\mathop {\pi }\limits ^{\leadsto
									}$$ with quantifiers, arrays, and bit-vectors.

2 Our new technique discovered bugs in 10 of the benchmarks that had been labelled safe. Satabs timed out before identifying these bugs.

3 Unfortunately, loop detection in Satabs is neither available nor maintained anymore.

## References

[1] Ball T, Cook B, Levin V, Rajamani SK (2004) Slam and static driver verifier: technology transfer of formal methods inside Microsoft. In: IFM, LNCS, vol 2999. Springer, New York

[2] Beyer D (2014) Status report on software verification—(competition summary SV-COMP 2014). In: TACAS, LNCS, vol 8413. Springer, New York, pp 373–388

[3] Beyer D, Henzinger TA, Majumdar R, Rybalchenko A (2007) Path invariants. In: PLDI. ACM, pp 300–309

[4] Boigelot B (1999) Symbolic methods for exploring infinite state spaces. Ph.D. thesis, Université de Liège

[5] Clarke EM, Kroening D, Lerda F (2004) A tool for checking ANSI-C programs. In: TACAS. Springer, New York, pp 168–176

[6] Cook B, Podelski A, Rybalchenko A (2011). Proving program termination. Commun ACM.

[7] van Engelen R, Birch J, Gallivan K (2004) Array data dependence testing with the chains of recurrences algebra. In: IEEE Innovative architecture for future generation high-performance processors and systems, pp 70–81

[8] Finkel A, Leroux J (2002) How to compose Presburger-accelerations: applications to broadcast protocols. In: FST-TCS 2002, LNCS, vol 2556. Springer, New York, pp 145–156

[9] Handjieva M, Tzolovski S (1998) Refining static analyses by trace-based partitioning using control flow. In: SAS, LNCS, vol 1503. Springer, New York, pp 200–214

[10] Henzinger TA, Jhala R, Majumdar R, Sutre G (2002) Lazy abstraction. In: POPL. ACM, pp 58–70

[11] Hojjat H, Iosif R, Konecny F, Kuncak V, Ruemmer P (2012) Accelerating interpolants. In: ATVA, LNCS. Springer, New York 7561:197–202

[12] Jhala R, McMillan KL (2006) A practical and complete approach to predicate refinement. In: TACAS, LNCS, vol 3920. Springer, New York, pp 459–473

[13] Kovács L, Voronkov A (2009) Finding loop invariants for programs over arrays using a theorem prover. In: FASE, LNCS, vol 5503. Springer, New York, pp 470–485

[14] Kovács LI, Jebelean T (2005) An algorithm for automated generation of invariants for loops with conditionals. In: IEEE symposium on symbolic and numeric algorithms for scientific computing (SYNASC), pp 245–249

[15] Kroening D, Sharygina N, Tonetta S, Tsitovich A, Wintersteiger CM (2008) Loop summarization using abstract transformers. In: ATVA, LNCS, vol 5311. Springer, New York, pp 111–125

[16] Kroening D, Weissenbacher G (2006) Counterexamples with loops for predicate abstraction. In: CAV, LNCS, vol 4144. Springer, New York, pp 152–165

[17] Kroening D, Weissenbacher G (2010). Verification and falsification of programs with loops using
predicate abstraction. Form Asp Comput.

[18] Ku K, Hart TE, Chechik M, Lie D (2007) A buffer overflow benchmark for software model checkers. In: ASE. ACM, pp 389–392. doi:10.1145/1321631.1321691

[19] McMillan KL (2006) Lazy abstraction with interpolants. In: CAV, LNCS, vol 4144. Springer, New York, pp 123–136

[20] Nelson G (1989). A generalization of Dijkstra’s
calculus. TOPLAS.

[21] Sharma R, Dillig I, Dillig T, Aiken A (2011) Simplifying loop invariant generation using splitter predicates. In: CAV, LNCS, vol 6806. Springer, New York, pp 703–719

[22] Sinha N (2008) Symbolic program analysis using term rewriting and generalization. In: Formal methods in computer-aided design, pp 1–9

[23] Zima H, Chapman B (1991). Supercompilers for parallel and vector computers.

[24] Zuleger F, Gulwani S, Sinn M, Veith H (2011) Bound analysis of imperative programs with the size-change abstraction. In: SAS, LNCS, vol 6887. Springer, New York, pp 280–297

